# *Staphylococcus aureus* Biofilm-Associated Infections: Have We Found a Clinically Relevant Target?

**DOI:** 10.3390/microorganisms13040852

**Published:** 2025-04-09

**Authors:** Karen E. Beenken, Mark S. Smeltzer

**Affiliations:** 1 Department of Microbiology and Immunology, University of Arkansas for Medical Sciences, Little Rock, AR 72205, USA; beenkenkarene@uams.edu; 2 Department of Biochemistry and Molecular Biology, University of Arkansas for Medical Sciences, Little Rock, AR 72205, USA; 3 Department of Orthopaedic Surgery, University of Arkansas for Medical Sciences, Little Rock, AR 72205, USA

**Keywords:** *Staphylococcus aureus*, biofilm, regulation, agr, sarA, sigB, xerC, protease, osteomyelitis

## Abstract

*Staphylococcus aureus* is one of the most diverse bacterial pathogens. This is reflected in its ability to cause a wide array of infections and in genotypic and phenotypic differences between clinical isolates that extend beyond their antibiotic resistance status. Many *S. aureus* infections, including those involving indwelling medical devices, are therapeutically defined by the formation of a biofilm. This is reflected in the number of reports focusing on *S. aureus* biofilm formation and biofilm-associated infections. These infections are characterized by a level of intrinsic resistance that compromises conventional antibiotic therapy irrespective of acquired resistance, suggesting that an inhibitor of biofilm formation would have tremendous clinical value. Many reports have described large-scale screens aimed at identifying compounds that limit *S. aureus* biofilm formation, but relatively few examined whether the limitation was sufficient to overcome this intrinsic resistance. Similarly, while many of these reports examined the impact of putative inhibitors on *S. aureus* phenotypes, very few took a focused approach to identify and optimize an effective inhibitor of specific biofilm-associated targets. Such approaches are dependent on validating a target, hopefully one that is not restricted by the diversity of *S. aureus* as a bacterial pathogen. Rigorous biological validation of such a target would allow investigators to virtually screen vast chemical libraries to identify potential inhibitors that warrant further investigation based on their predicted function. Here, we summarize reports describing *S. aureus* regulatory loci implicated in biofilm formation to assess whether they are viable targets for the development of an anti-biofilm therapeutic strategy with an emphasis on whether *sarA* has been sufficiently validated to warrant consideration in this important clinical context.

## 1. Introduction

A PubMed search on 12 February 2025 using the phrase “staphylococcus aureus biofilm” identified 11,298 manuscripts. This reflects the importance of *Staphylococcus aureus* biofilms as a unique bacterial lifestyle that compromises the efficacy of host defenses and conventional antibiotic therapy [1,2,3,4,5,6]. Biofilms have classically been defined as aggregates of bacteria attached to a surface and encased in a self-produced extracellular matrix [7]. In medicine, this surface may be biotic or abiotic in the form of an indwelling medical device. More recent definitions include “non-attached biofilm aggregates” [8] and “staphylococcal abscess communities” (SACs) [9], which are not attached to a surface but also exhibit the tolerance to conventional antibiotics that clinically defines a biofilm. Some antibiotics are more effective than others in the context of a biofilm [10,11,12,13], but this tolerance confers a clinically relevant level of intrinsic resistance to even the most effective of antibiotics. The mechanistic basis for this is multi-factorial but best defined by reduced metabolic activity in biofilm-associated cells leading to the development of small colony variants (SCVs) and persister cells [3,6,14,15,16,17,18]. This is because the efficacy of essentially all antibiotics is dependent on metabolic activity in the targeted bacterial cells, and the few examples in which this is not the case (e.g., colistin) are only used systemically as a last resort owing to potential toxicity. This suggests that conventional approaches to antibiotic discovery and development are unlikely to overcome the therapeutic recalcitrance of *S. aureus* biofilm-associated infections. This suggests in turn that an anti-biofilm strategy is needed that can be used to help overcome the therapeutic recalcitrance of *S. aureus* biofilm-associated infections.

The clinical importance of biofilm-associated intrinsic resistance is reflected in the many reports focused on identifying factors that limit *S. aureus* biofilm formation. The number of these reports makes it important to put the massive amount of literature into perspective. Many studies focused on large-scale screens aimed at identifying compounds that limit biofilm formation, while others focused on gaining a better understanding of the mechanistic basis of *S. aureus* biofilm formation to allow a targeted approach. Using either approach, it is important to recognize the diversity of *S. aureus* as a pathogen, particularly with respect to its virulence factor repertoire and the regulatory circuits that control production of these virulence factors. Indeed, strain-dependent differences that impact biofilm formation and the pathogenesis of biofilm-associated infections have been documented [19,20,21]. Additionally, few studies that assessed whether specific compounds limit biofilm formation also assessed whether the limitation was correlated with increased antibiotic susceptibility, thus leaving a critical gap in our knowledge. The same is true of studies evaluating *S. aureus* genes involved in biofilm formation, with many genes having been implicated but relatively few having been evaluated in the defining clinical context of antibiotic susceptibility, particularly under in vivo conditions [10,19,22].

Among the most therapeutically recalcitrant of *S. aureus* biofilm-associated infections are those involving bone and indwelling orthopaedic devices. Treatment of these infections requires long-term systemic antibiotic therapy, often accompanied by surgical debridement [23]. Having gained direct access to the infection site during debridement, systemic therapy is often augmented by local, matrix-based antibiotic delivery aimed at achieving a concentration of antibiotic at the site of infection that is high enough to overcome the intrinsic resistance of SCVs and persister cells while avoiding systemic toxicity [24]. However, even after such intensive medical and surgical intervention, the recurrence rate can exceed 20% [23,25,26,27]. The cost of this is astronomical and rapidly increasing owing to the need to maintain functional mobility in an increasingly aging population [28,29]. Orthopaedic infections are not the only example of a biofilm-associated infection, but these alone warrant an effort to develop an effective anti-biofilm strategy. Most bacterial pathogens form biofilms, and many can cause orthopaedic infections, but the most common cause, and the cause of the most severe forms of infection characterized by extensive cortical bone destruction, is *S. aureus* [30,31,32].

Based on this, we have placed an emphasis on overcoming the therapeutic recalcitrance of *S. aureus* orthopaedic infections. While biofilm formation is a critical feature of these infections, other factors also contribute to this recalcitrance. Among these are the cortical bone destruction that compromises the local vasculature to a degree that limits the efficacy of systemic antibiotic delivery, invasion of *S. aureus* into the protective niche of the osteocyte lacuno-canalicular network (OLCN), and invasion of host cells including osteoblasts and osteoclasts [3]. We have confirmed the extensive bone loss and biofilm formation in both mouse and rabbit models of osteomyelitis [20,33], while others have convincingly demonstrated invasion of the OLCN and even identified *S. aureus* mutants that are compromised in this respect [34]. There is considerable in vitro evidence of *S. aureus* internalization by osteoblasts and osteoclasts [35,36,37], but in our opinion, compelling in vivo evidence that this internalization contributes to the therapeutic recalcitrance of these infections is lacking.

Of these phenotypes, biofilm formation is the easiest to assess in a high-throughput manner. Thus, a common experimental approach has been to screen small-molecule libraries for compounds that limit biofilm formation and/or eradicate an established biofilm [38]. The alternative to such screens is to use a targeted approach to identify key mechanistic factors that contribute to *S. aureus* biofilm formation [39]. The latter has the advantage of facilitating functional screens of vast chemical libraries at minimal expense based on predicted interactions between potential inhibitors and their targets. However, given the cost of drug discovery and development [40], it is imperative that such an approach starts with a validated target. *S. aureus* produces a remarkable array of virulence factors, many of which are functionally redundant. For this reason, much of the targeted research effort has focused on regulatory loci rather than individual virulence factors, with the accessory gene regulator (*agr*) and the staphylococcal accessory regulator (*sarA*) receiving the most attention [41,42,43,44,45,46,47,48,49,50,51]. Both *sarA* and *agr* mutants also exhibit reduced virulence in murine models of bacteremia and osteomyelitis [20,52,53,54,55,56]. Studies investigating these regulatory loci can also help identify critical virulence factors based on correlations between their abundance in *sarA* and *agr* mutants and relative virulence [57].

## 2. Impact of *agr* and *sarA*

The *agr* locus encodes a quorum sensing system that plays a central regulatory role in *S. aureus.* As assessed in vitro, this is broadly characterized by a shift from the production of surface-associated virulence factors during the exponential growth phase to the production of extracellular virulence factors during post-exponential growth [58]. The two *agr*-encoded effector molecules are the AgrA response regulator and a regulatory RNA designated RNAIII [22,50,51]. Once phosphorylated, AgrA enhances transcription from the divergent promoter encoding RNAIII, with RNAIII then modulating production of key virulence factors like protein A and α-toxin at a translational level [59,60,61]. The only other known *S. aureus* virulence factors produced under the direct regulatory control of AgrA are the phenol-soluble modulins (PSMs) [62].

The *sarA* locus encodes a 15 kDa DNA-binding protein that enhances expression of the *agr* operon, suggesting that *sarA* is upstream of *agr* and functions through an *agr*-dependent pathway [63,64,65,66]. However, it has become increasingly clear that *sarA* also functions through an *agr*-independent pathway. For example, mutation of *sarA* results in increased protease production, while mutation of *agr* has the opposite effect [57]. Mutation of *sarA* also limits biofilm formation in diverse clinical isolates of *S. aureus*, while mutation of *agr* has little effect or, in strains that express *agr* at high levels, enhances biofilm formation [67,68]. This indicates that the regulatory functions of *sarA* that are most relevant in the context of biofilm formation and biofilm-associated infections are mediated via an *agr*-independent pathway. Moreover, mutation of *sarA* in the USA300 strain LAC limits virulence in a murine osteomyelitis model to a greater extent than mutation of *agr* [52], and this would not be expected if the key regulatory functions of *sarA* were mediated through *agr*.

From a therapeutic point of view, it is also important to note that spontaneous *agr* mutants arise in vivo [69,70,71,72,73,74]. Under in vitro conditions, these mutants ultimately become the dominant subpopulation. It has even been proposed that such mutants promote the transition from acute to chronic infection [71]. This suggests that inhibitors of *agr* might have the unintended consequence of promoting this transition. To our knowledge, there are no reports describing spontaneous *sarA* mutants being isolated from patients. Moreover, the impact of mutating *sarA* on biofilm formation and the pathogenesis of osteomyelitis is evident even in an *agr* mutant, thus suggesting that a *sarA* inhibitor would retain its therapeutic efficacy even in the context of such spontaneous *agr* mutants [52].

## 3. Impact on Biofilm-Associated Intrinsic Resistance

Mutation of *sarA* limits biofilm formation to a degree that can be correlated with increased susceptibility to functionally diverse classes of antibiotics [10,12,75,76]. Among these are vancomycin, linezolid, ceftaroline and daptomycin, all of which are active against methicillin-resistant *S. aureus* (MRSA). As assessed using an in vitro model of catheter-associated biofilm formation, daptomycin and ceftaroline, a fifth-generation cephalosporin, are more active than oxacillin and vancomycin, which are commonly used to treat infections caused by methicillin-susceptible and methicillin-resistant *S. aureus*, respectively [17]. Most importantly, mutation of *sarA* increased the susceptibility of the methicillin-susceptible strain UAMS-1 and the methicillin-resistant strain LAC to both ceftaroline and daptomycin [10]. Mutation of *sarA* also results in increased susceptibility to ciprofloxacin and vancomycin, at least in vancomycin-intermediate strains [77]. Mutation of *sigB* also limits biofilm formation and increases the susceptibility of *S. aureus* to multiple antibiotics, which is consistent with the observation that expression of *sarA* is *sigB*-dependent [78,79,80,81], but mutation of *sarA* has a greater impact on these phenotypes than mutation of *sigB* [10,19,82].

At the same time, *sigB* plays a critical role in the development of SCVs and the transition from acute to chronic infection [80,81,82,83,84], suggesting that it may be a viable if not preferred target for therapeutic intervention in the context of chronic, biofilm-associated *S. aureus* infections. However, while expression of *sarA* is decreased in a *sigB* mutant, expression of *agr* is increased, thus suggesting that targeting *sigB* would have the adverse consequence of increasing toxin production [22,83,84]. Mutation of *sarA* also results in increased production of critical toxins, but the abundance of full-length functional forms of these toxins is limited in *sarA* mutants owing to protease-mediated degradation [20,52].

## 4. Impact on Oxacillin Resistance

A primary therapeutic consideration in the treatment of *S. aureus* infections is whether the causative strain is resistant to the methicillin class of antibiotics (e.g., oxacillin). This is because MRSA are often resistant to multiple antibiotics including all β-lactams other than fifth-generation cephalosporins [85,86]. In most cases, the impact of mutating *sarA* on the intrinsic resistance of *S. aureus* biofilms cannot be attributed to changes in the minimum inhibitory concentration (MIC), but mutation of *sarA* in MRSA does reduce the MIC for oxacillin to a level at or below the susceptibility breakpoint of ≤2 μg/mL [57,87]. In the MRSA strains LAC, mutation of *agr* increases oxacillin susceptibility even more than mutation of *sarA* [57], but the impact of mutating *sarA* on both intrinsic and acquired susceptibility suggests that an effective inhibitor of *sarA* could be used in combination with existing antibiotics to prophylactic and therapeutic advantage in biofilm-associated *S. aureus* infections.

## 5. Biofilm Inhibitors as Therapeutic Options

Conventional antibiotics have bacterial targets that are essential to growth (bacteriostatic) or survival (bactericidal). There is considerable debate about whether non-essential targets are also viable therapeutic options, but enthusiasm for such strategies has increased in the current era of rapidly increasing antibiotic resistance and rapidly diminishing antibiotic discovery [88,89,90,91]. Biofilm formation is not an essential phenotype, so it is difficult to envision a therapeutic approach that would rely solely on an anti-biofilm agent, with the more likely possibility being that such an agent would be used in combination with conventional antibiotics. Whether such strategies are economically viable, particularly when targeting a single bacterial pathogen, depends on the ability to demonstrate a definitive cause-and-effect relationship between that pathogen and an important clinical problem. It is also important that the therapeutic efficacy of such an inhibitor be evident in the context of the diversity among clinical isolates of the targeted pathogen including their acquired antibiotic-resistance status.

## 6. Diversity in *S. aureus*

Diversity in *S. aureus* is reflected in several typing schemes including multi-locus sequence typing (MLST), which is based on genotypic differences in each of 7 housekeeping genes [92]. This allows for the grouping of clinical isolates into sequence types (ST), and the grouping of related sequence types into clonal complexes (CC) [93]. The Centers for Disease Control and Prevention (CDC) use pulsed-field gel electrophoresis (PFGE) to categorize strains into lineages designated by the prefix “USA” (e.g., USA100), with USA300 largely defining the emergence of community-associated MRSA (CA-MRSA) strains exemplified by the Los Angeles County (LAC) clone [94]. The clinical prominence of USA300 CA-MRSA isolates is why a plasmid-free derivative of LAC (JE2) was used to generate the Nebraska Transposon Mutant Library (NTML), which contains mutants with transposon insertions in 1952 non-essential genes [95].

While the clinical prominence of CA-MRSA strains demands attention, it does not preclude the need to consider other lineages. Our own studies focusing on osteomyelitis were initiated using the methicillin-susceptible CC30, USA200 isolate UAMS-1 because it was isolated directly from the bone of a patient undergoing surgical debridement [54,96]. Interestingly, debridement was required despite the fact that UAMS-1 (ATCC 49230) is sensitive to all antibiotics other than penicillin. UAMS-1 was subsequently used to develop and characterize murine and rabbit osteomyelitis models [33,52]. Characteristics that distinguish UAMS-1 from LAC include the presence of the collagen-binding adhesion gene *cna*, the presence of the *tst* gene encoding toxic shock syndrome toxin-1 (TSST-1), the absence of the genes (*lukF*, *lukS*) encoding components of the bicomponent Panton–Valentine leucocidin (PVL), the absence of one of two genes encoding fibronectin-binding proteins (*fnbB*), the absence of the *sarA* homologs *sarT* and *sarU*, and the absence of any form of the staphylococcal chromosome cassette that includes *mecA* (SCC*mec*) [96]. These differences are important because many have been implicated in biofilm formation and the pathogenesis of biofilm-associated infections including osteomyelitis [33,96,97,98,99].

UAMS-1 also does not produce α-toxin owing to a nonsense mutation in the corresponding gene (*hla*) [100]. There is a report concluding that α-toxin is required for biofilm formation in *S. aureus* [101], but this is called into question by the observation that UAMS-1 forms a robust biofilm both in vitro and in vivo [67]. Moreover, it has been demonstrated that non-hemolytic variants of *S. aureus* that do not produce α-toxin arise in biofilms and ultimately become the predominant subpopulation [74]. This was attributed to limited expression of *agr* and *sarU*, an *agr*-activating regulatory element that is absent in UAMS-1. However, the more important point is that LAC and UAMS-1 are distinct by comparison to each other to a degree that makes them representative of the diversity among clinical isolates of *S. aureus*. Although LAC expresses *agr* at higher levels than UAMS-1, LAC and UAMS-1 form comparable biofilms. Both are also virulent in diverse forms of *S. aureus* infection including bacteremia and osteomyelitis, although LAC is demonstrably more virulent than UAMS-1 in both models [21,53,55,102].

The evolutionary distance between USA300 strains like LAC and USA200 strains like UAMS-1 is well established [97,103], but this is not to say that LAC and UAMS-1 fully encompass all of the genotypic and phenotypic diversity in *S. aureus*. Indeed, there is genetic variability even among clonal lineages including USA300. However, the USA300 core genome is similar, and the complete genome sequence for a representative strain (FPR3757) is available. The sequence of the plasmid-free strain JE2, which was derived from LAC and used to generate the NTMLNTML library, is also available [103,104,105,106,107], as is the genome sequence of UAMS-1 and other USA200 isolates like MRSA252 [103,108].

## 7. Biofilm Formation as a Function of Methicillin Resistance

Methicillin resistance is not definitively associated with any specific ST, CC, or USA lineage, but there are reports concluding that the mechanism of biofilm formation differs between MRSA and MSSA. Specifically, it has been proposed that biofilm formation in MRSA is dependent on surface-associated proteins, while in MSSA, it is dependent on the *ica*-encoded polysaccharide intercellular adhesin (PIA) [109,110,111]. This suggests multiple inhibitors might be required to combat MRSA vs. MSSA biofilm-associated infections. However, mutation of *sarA* limits biofilm formation more than mutation of any other *S. aureus* regulatory locus examined to date, and it does so in diverse clonal lineages including those represented by the MRSA strain LAC and the MSSA strain UAMS-1 [10,12,19,112,113]. In fact, the only exceptions we have identified are RN6390 and Newman, which are commonly used laboratory strains with mutations in other regulatory loci that limit biofilm formation, thereby masking the phenotypic impact of mutation *sarA* on biofilm formation. Indeed, if these defects are repaired, the biofilm-deficient phenotype of the isogenic *sarA* mutants is evident to the same degree we have observed in every clinical isolate we have examined to date [112,113,114,115].

Specifically, RN6390 has a mutation in *rsbU*, which is part of the *sigB* regulon, and consequently expresses *agr* at high levels [116]. RN6390 does not form a robust biofilm even under conditions optimized to promote biofilm formation in vitro [67]. Repair of this defect enhances biofilm formation [117]. Mutation of *agr* also enhances biofilm formation in RN6390 [67]. One possible explanation for the biofilm deficient phenotype of RN6390 and the restoration of biofilm formation in an RN6390 *agr* mutant is that AgrA enhances production of PSMs, which act as surfactants and serve as a regulated means of dispersal from an established biofilm [62,118]. However, the more important consideration in the context of this discussion is that the biofilm-positive phenotype of an RN6390 *agr* mutant is reversed in a *sarA/agr* mutant [68]. This demonstrates that the impact of *sarA* on biofilm formation is epistatic to *agr* in the specific context of biofilm formation.

Similarly, Newman has a mutation in *saeS* that results in its constitutive activation as well as mutations that truncate the fibronectin-binding proteins such that they are not anchored to the cell wall [119,120]. These defects limit the capacity of Newman to form a biofilm, and biofilm formation is enhanced if either or both are repaired, in which case mutation of *sarA* limits biofilm formation to a degree comparable to other clinical isolates [114,121]. The cumulative results investigating these exceptions also point to key elements of the *sarA* regulatory paradigm as it relates to biofilm formation, specifically the ability of SarA to repress protease production in an *agr*-independent manner [52,57,68].

## 8. Why *sarA* Mutants Do Not Form a Biofilm

The fact that mutation of *sarA* results in increased protease production and decreased biofilm formation suggests a cause-and-effect relationship, and this is confirmed by the observation that biofilm formation is fully restored in MSSA and MRSA *sarA* mutants by eliminating their ability to produce the extracellular proteases aureolysin, ScpA, SspA, SspB, and SplA-F [20,102,112,114,122,123,124]. This would not be expected if the primary mechanism of biofilm formation in MSSA strains is mediated by PIA. Indeed, mutation of the *ica* operon, which encodes the enzymes necessary to produce PIA, in the MSSA strain UAMS-1 limits biofilm formation, but only to a modest extent by comparison to mutation of *sarA* [68,112,123].

Mutation of *sarA* also results in increased nuclease production [125], and extracellular DNA (eDNA) has been implicated as a key component of the biofilm extracellular matrix in both MSSA and MRSA [126]. This suggests that degradation of eDNA might also contribute to the biofilm-deficient phenotype of *sarA* mutants. We confirmed that mutation of the *S. aureus* genes encoding its primary extracellular and surface-associated nucleases, specifically *nuc1* and *nuc2*, enhances biofilm formation in vitro [123,125]. However, eliminating nuclease production did not enhance biofilm formation in vivo in a murine model of catheter-associated biofilm formation [125]. These results do not contradict the conclusion that eDNA contributes to biofilm formation, or that PIA contributes more in MSSA than in MRSA, but do demonstrate that the impact of PIA and eDNA is overwhelmed by the increased production of extracellular proteases in *sarA* mutants. This suggests a common intervention point that could be exploited to prophylactic and/or therapeutic advantage in *S. aureus* biofilm-associated infections.

## 9. Why Increased Protease Production Limits Biofilm Formation

Staphylococcal protein A (Spa) and the fibronectin-binding proteins FnbA and FnbB are among the *S. aureus* surface proteins that contribute to biofilm formation [127,128]. The MSSA osteomyelitis isolate UAMS-1 encodes FnbA but not FnbB, the corresponding gene for which (*fnbB*) is part of a chromosomal “region of difference” (RD5) that also includes *sarT* and *sarU* [129]. Mutation of *fnbA* and *spa* in UAMS-1 limits biofilm formation [112], and both FnbA and Spa are absent or present in dramatically reduced amounts in *sarA* mutants owing to protease-mediated degradation. This suggests that the protease-mediated degradation of Spa and FnbA/FnbB contributes to the biofilm-deficient phenotype of *sarA* mutants [52,53,57,114]. However, as with *ica* and *nuc1/nuc2* mutants, the limitation observed in an *fnbA/spa* mutant is less than that observed in an isogenic *sarA* mutant, suggesting that the impact of mutating *sarA* also extends beyond these specific biofilm-associated proteins [112].

Proteomics comparisons confirmed that the increased production of extracellular proteases in *sarA* mutants compromises much of the *S. aureus* proteome [57,115,130]. Indeed, of 1039 proteins identified in conditioned medium (CM) from an overnight (16 h) stationary-phase culture of the MRSA strain LAC, only 139 (13.4%) were detected in CM from the isogenic *sarA* mutant, while 870 (83.7%) were detected in a protease-deficient *sarA* mutant [57]. Thus, the ability to detect 731 of 1039 *S. aureus* proteins (70.4%) in CM from *sarA* mutants was compromised owing to protease-mediated degradation. This demonstrates the importance of *sarA* in limiting extracellular protease production as a means of post-translationally remodeling the *S. aureus* proteome, with mutation of *sarA* resulting in increased protease production to an extent that can be correlated with the decreased abundance of many *S. aureus* proteins other than Spa and FnbA/FnbB that may also contribute to biofilm formation and maintenance of the biofilm lifestyle.

## 10. Impact of *sarA* on Other Critical Phenotypes

The global impact of increased protease production on the proteome of *sarA* mutants suggests that the phenotypic impact would extend beyond biofilm formation. This is consistent with the observation that mutation of *sarA* also limits cortical bone destruction in our murine osteomyelitis model as well as virulence in a murine model of acute bacteremia [56,63]. It is also consistent with the observation that the increased protease production observed in *sarA* mutants limits the abundance of full-length and presumably functional forms of multiple cytolytic toxins including α-toxin, PSMs, and both LukF and LukS, which are the two components of the bicomponent cytotoxin PVL [52,55]. This is likely to be particularly relevant in limiting the cortical bone destruction observed in mice infected with *sarA* mutants. Indeed, CM from LAC and UAMS-1 is cytolytic for osteoblasts and osteoclasts in vitro, and this cytotoxicity is eliminated in CM from *sarA* mutants owing to protease-mediated degradation [52,53]. Osteoblasts and osteoclasts are key cells involved in normal bone remodeling, and both have been implicated in the pathogenesis of *S. aureus* bone infection [131,132]. Additionally, Spa contributes to cortical bone destruction through increased osteoclastogenesis, and an anti-Spa antibody delivered by intraperitoneal injection was shown to prevent excessive inflammatory responses in the bone by limiting the response of osteoclasts to RANKL [133]. Studies with Denosumab (Prolia, Xgeva), a monoclonal antibody that inhibits RANKL signaling and is used to limit bone loss in osteoporosis, hypercalcemia, and bone cancer, demonstrated a lack of osteoclasts in infected bone and reduced cortical bone destruction, thus confirming a critical role for osteoclasts in the pathogenesis of *S. aureus* osteomyelitis [134]. This is consistent with the hypothesis that the absence of Spa in CM from *sarA* mutants limits osteoclastogenesis and cortical bone loss, which may be particularly relevant in strains like UAMS-1 that do not produce α-toxin or PVL and produce PSMs at relatively low levels, but also produce Spa at relatively high levels [52,53,55].

Mutation of *sarA* also results in reduced intracellular survival in neutrophils, which are the primary front-line defense against *S. aureus* infection [135]. Interestingly, unlike *sigB* and *agr*, *sarA* was not found to contribute to intracellular survival in macrophages [136]. Conversely, PSMs are intracellular as well as extracellular toxins and have been shown to kill infected osteoblasts [137]. PSMs are present in reduced amounts in *sarA* mutants owing to protease-mediated degradation [55] and this suggests that *sarA* mutants may exhibit enhanced survival in osteoblasts. This could be particularly critical in the pathogenesis of osteomyelitis as suggested by the observation that *S. aureus* isolates from chronic osteomyelitis have a high capacity for host cell invasion [138]. However, this must be interpreted with caution given that protease-mediated degradation also limits the abundance of specific *S. aureus* virulence factors that promote internalization by non-phagocytic cells, particularly the fibronectin-binding proteins FnbA and FnbB [139,140,141].

## 11. Protease-Mediated Post-Translational Regulation (Figure 1)

Regulation in *S. aureus* is dynamic, complex, and highly interactive as evidenced by the observation that *sarA* has both *agr*-dependent and *agr*-independent regulatory functions. Studies investigating these interactions have focused almost exclusively on transcriptional or, in the case of RNAIII, post-transcriptional regulation [142,143,144,145]. However, it is increasingly evident that extracellular proteases play a critical post-translational role in fine tuning the *S. aureus* proteome in a fashion that impacts biofilm formation and virulence in both acute and chronic models of *S. aureus* infection [20,53,56,124,146]. The importance of this is reflected in the number of regulatory loci implicated in protease production (see below). It is also reflected in the fact that eliminating protease production increases virulence, while overproducing proteases decreases virulence, both owing to the impact of proteases on the *S. aureus* proteome [52,147,148]. Most manuscripts summarizing regulatory circuits in *S. aureus* do not reflect the importance of this protease-mediated post-translational regulation [142,143,144,145], and those that highlight the impact of *sarA* on protease production do not reflect the phenotypic impact of the increased production of these proteases [22].

**Figure 1:**
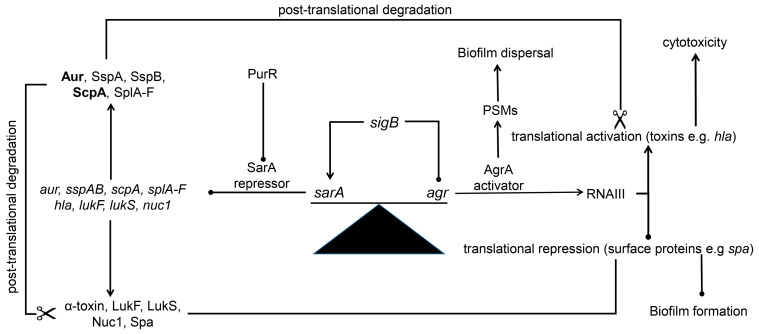
**Schematic illustrating the critical balance between *sarA* and *agr* regulatory functions.** The model proposes that the critical regulatory functions of *sarA* are independent of any direct interaction with *agr* and primarily defined by the ability of SarA to directly repress the production of critical extracellular proteases, most notably aureolysin and ScpA (**bold**). It also proposes that SarA functions primarily as a transcriptional repressor with respect to other *S.aureus* exoproteins, but that the phenotypic impact of this is limited owing to increased protease production. The model emphasizes the critical need to balance the regulatory functions of *sarA* and *agr*, and the potential role of *sigB* in this regard. In this model, the key regulatory functions of *sarA* and *agr* are independent of each other at a transcriptional level, which in itself is a paradigm shift, but nevertheless interdependent in that the reduced abundance of extracellular proteins, including proteases, in *agr* mutants occurs at a translational level owing to the absence of RNAIII, with this effect being moderated by increased transcription of the corresponding genes in an isogenic *sarA* mutant.

Indeed, the cumulative data demonstrate that the primary regulatory functions of *sarA* and *agr* are independent of each other with respect to the impact of one on expression of the other, particularly in the context of biofilm formation, but we propose that the impact of one is in fact dependent on the functional status of the other. For example, analysis of α-toxin production in *sarA*, *agr*, and *sarA/agr* mutants and their isogenic protease-deficient derivatives demonstrated that *sarA* represses the production of α-toxin, but the increase in α-toxin abundance is phenotypically irrelevant owing to protease-mediated degradation. In the absence of *agr*, α-toxin is not produced owing to the absence of RNAIII and the resulting defect in translation, while in a *sarA/agr* mutant, the α-toxin phenotype is intermediate between these extremes, perhaps because transcription of *hla* is greatly increased owing to the absence of *sarA*, but translation remains stunted owing to the absence of *agr* or, more specifically, RNAIII. This hypothesis is consistent with the recognized post-transcriptional role of RNAIII in translation of *hla* mRNA [61]. Such a regulatory scenario could also account for the relative impact of these two critical loci on the production of other extracellular proteins including proteases. Indeed, protease production is also intermediate in a *sarA/agr* mutant by comparison to isogenic *sarA* and *agr* mutants but sufficient to restrict the abundance of *S. aureus* proteins to a degree that can be correlated with reduced virulence [52]. In this scenario, the reduced abundance of extracellular proteins, including proteases, in *agr* mutants occurs at a translational level owing to the absence of RNAIII, with this effect being moderated by increased transcription of the corresponding genes in an isogenic *sarA* mutant.

## 12. How *sarA* Limits Protease Production

The 10 primary extracellular proteases produced by *S. aureus* are aureolysin (Aur), staphopain A (ScpA), serine protease A (V8 protease, SspA), cysteine protease B (Staphopain B, SspB), and the serine protease-like proteases (SplA-F). These are encoded singly (*aur*, *scpA*) or as part of an operon (*sspAB*, *splA-F*), thus constituting four transcriptional units [149]. SarA binds DNA upstream of all four of these transcriptional units, and expression of all four is increased in *sarA* mutants. SELEX (systematic evolution of ligands by exponential enrichment) identified a 7 bp sequence (ATTTTAT) as a putative SarA binding site [150]. This is of little predictive value as *S. aureus* has an AT-rich genome, but potential SarA binding sites (ATTTTAA, TTTTATT, ATATTTT or ATTTTTT) were identified upstream of all protease genes/operons other than *splA-F* [122].

These putative binding sites are all located upstream of the predicted promoter elements, and progressive deletion of these upstream regions confirmed increased expression from all four promoters in a wild-type strain lacking these upstream regions. This was true even with *splA-F*, which likely reflects the sequence ambiguity in SarA binding sites. Thus, the protease phenotype of a *sarA* mutant can be replicated in a wild-type strain by eliminating the regions containing these putative binding sites. SarA itself was captured using DNA baits derived from these regions [122], suggesting that there are functional SarA binding sites upstream of all four protease promoters, and that SarA functions as a direct transcriptional repressor of all ten extracellular proteases.

Using a mass-based proteomics approach that distinguishes between full-length and truncated proteins, we also demonstrated that, with the exception of SspB, the abundance of these proteases is increased in CM from overnight cultures of *sarA* mutants [57]. However, when the size restriction is removed, the total amount of SspB in either a truncated or full-length form is also increased. These results are consistent with the conclusion that transcription of the genes encoding extracellular proteases is increased in *sarA* mutants via the direct binding of SarA and that this results in a corresponding increase in the overall abundance of all 10 primary *S. aureus* extracellular proteases.

## 13. Relative Importance of Individual Proteases

Protease production in *S. aureus* is driven in part by a cascade in which aureolysin activates SspA and SspA in turn activates SspB [149]. This complicates efforts to assess the relative impact of specific proteases. However, purified aureolysin was found to limit biofilm formation in 14 of 15 methicillin-resistant isolates and 11 of 15 methicillin-susceptible isolates, suggesting that aureolylsin itself plays an important role [112]. Studies demonstrating that a derivative of LAC unable to produce any extracellular protease was hypervirulent in a sepsis model that also found that the production of aureolysin and ScpA in combination with each other reversed this phenotype [148]. Independent studies investigating the impact of different proteases in defining the reduced virulence of *sarA* mutants also demonstrated that the inability to produce these same two proteases had the greatest impact on restoring the virulence of both LAC and UAMS-1 *sarA* mutants in osteomyelitis [20]. Biofilm formation was also restored in LAC and UAMS-1 *sarA/aur/scpA* mutants, while mutation of *aur* had the greatest impact on the cytotoxicity of CM from *sarA* mutants to mammalian cells, particularly with CM from the more cytotoxic strain LAC. However, concomitant mutation of *sspAB* was required to mimic the overall proteome profile of *sarA* mutants unable to produce any extracellular protease [20].

## 14. The Impact of Other *S. aureus* Regulatory Loci

While the cumulative data support the hypothesis that *sarA* may be a viable therapeutic target to combat biofilm-associated *S. aureus* infections, this does not mean that *sarA* is the only or even the best target, particularly since many other *S. aureus* regulatory mutants exhibit altered biofilm formation and changes in protease production. Indeed, a screen of mutants in the Nebraska Transposon Mutant Library (NTML) using milk agar plates identified 62 mutants with altered protease activity, 12 of which exhibited increased activity [95]. These included mutations in *sarA*, *saeR*, and multiple genes in the *sigB* regulon (*rsbU*, *rsbV*, *rsbW*, and *rpoF*). Mutation of *sigB* also limits biofilm formation and virulence in a murine sepsis model to a degree approaching that observed in an isogenic *sarA* mutant [56,103], and the limited capacity of a *sigB* mutant to form a biofilm is sufficient to increase antibiotic susceptibility in both LAC and UAMS-1 [10]. These phenotypes are also largely restored in *sigB* mutants by eliminating protease production. However, mutation of *sigB* has a limited impact on protease production and biofilm formation by comparison to mutation of *sarA*. One possible explanation for this is that *sigB* is upstream of *sarA*, thus making SarA the functional response regulator at least in the context of protease production, but this does not preclude *sigB* as a viable target, particularly given its role in SCV formation and the transition from acute to chronic infection [80,81,82,83,84].

A *xerC* mutant also exhibited increased protease production in the NTML screen [95], and we independently confirmed this observation in *xerC* mutants generated in both LAC and UAMS-1 [151]. Like *sarA* and *sigB* mutants, LAC and UAMS-1 *xerC* mutants are also attenuated in a murine sepsis model. Whether this is true in a biofilm-associated infection like osteomyelitis remains to be determined, as does the impact of increased protease production, particularly since *xerC* also impacts the production of *S. aureus* virulence factors, specifically α-toxin and Spa, through an *agr*-dependent mechanism. These direct comparisons suggest that biofilm formation is limited in *xerC* mutants owing to *agr*-independent regulation, while virulence in acute infection is dependent on the impact of *xerC* on *agr* [151]. Moreover, biofilm formation was enhanced in a *xerC* mutant unable to produce extracellular proteases, albeit to a lesser extent than was observed in *sarA* and *sigB* mutants, thus further demonstrating that the impact of these mutants on biofilm formation occurs via an *agr*-independent mechanism mediated at least in part by the increased production of extracellular proteases.

Conversely, while *sarA* and *sigB* also have *agr*-dependent regulatory functions, the cumulative data would suggest that the impact of *xerC* in this regard may exceed that of either of these other regulatory loci. This suggests that *xerC* could offer a therapeutic advantage in diverse forms of *S. aureus* infection. Moreover, XerC is a tyrosine recombinase, and *xerC* was recently identified in a screen for mutants in DNA repair pathways that are required for the SOS response in *S. aureus* [152]. A *xerC* mutant also exhibited increased susceptibility to ciprofloxacin and other DNA-damaging agents, as well as reduced survival in whole blood from healthy donors [152] and increased sensitivity to daptomycin and ceftaroline in the context of established biofilms formed by both UAMS-1 and LAC [151].

Interestingly, *SarA* and the histone-like protein HU were shown to be among 299 proteins associated with the *S. aureus* nucleoid, particularly during periods of oxidative stress [153]. Somewhat anecdotally, we found that when using phage-mediated transduction to generate multiple mutations in the same strain, when one of these mutations is in *sarA,* it is more efficient to move the *sarA* mutation last. This is consistent with the demonstration that SarA has functions related to bacteriophage integration and excision [154]. Like *xerC*, mutation of *sarA* also increases susceptibility to ciprofloxacin [77].

Finally, an *S. aureus mgrA* mutant exhibits an enhanced capacity to select for SCVs, perhaps owing to an increased rate of mutagenesis [155]. Genome sequencing of the resulting SCVs found that one of the potentially contributing factors was a single nucleotide polymorphism (SNP) in *xerC*. The other SNP identified in this screen was in *glmM*, which encodes phosphoglucosamine mutase [155]. Thus, inhibition of *xerC* could have the adverse consequence of promoting development of SCVs. These conflicting predictors of the pathogenesis and therapeutic recalcitrance of *xerC* mutants emphasize the need to assess the relative impact of *xerC*, *sarA* and *sigB* mutants in *S. aureus* biofilm-associated infection.

## 15. Another Side of the *sarA* Protease Paradigm

In contrast to *sarA*, *sigB* and *xerC* mutants, the NTML screen found that mutation of *purR* results in decreased protease production [95], and it was subsequently demonstrated that a *purR* mutant exhibited increased virulence in a murine sepsis model [156]. Decreased protease production in a *purR* mutant is correlated with the increased abundance of α-toxin, Spa, FnbA/FnbB, and SarA itself. These phenotypes are correlated with an increased capacity to form a biofilm and increased virulence in our osteomyelitis model [157]. Mutation of *sarA* has the opposite effect on these phenotypes, and the phenotypes of *sarA*/*purR* mutants were comparable to those of *sarA* mutants. This suggests that a critical component defining the increased virulence of a *purR* mutant is the enhanced production of SarA [157]. Increased expression of *sarA* in a *purR* mutant was independently confirmed in vivo in cardiac vegetations, further suggesting that *sarA* plays a critical role in vivo in balancing the abundance of *S. aureus* virulence factors and central metabolic elements like those involved in purine biosynthesis [158]. Mutation of *purR* also resulted in reduced susceptibility to vancomycin, suggesting that any therapeutic strategy targeting *purR* would require increased rather than decreased expression. Nevertheless, these results are consistent with the hypothesis that dysregulation of extracellular proteases, whether increased or decreased, plays a key role in the pathogenesis of *S. aureus* biofilm-associated infection.

## 16. Regulatory Loci Not Identified in the NTML Screen

More targeted mutagenesis studies have led to the identification of additional *S. aureus* regulatory loci that impact protease production but were not identified in the NTML screen. These include *mgrA*, *sarR*, *sarS*, *sarZ*, and *rot*, all of which encode proteins that, like SarA, were captured in DNA-binding experiments using protease-associated promoter regions as bait [122]. Direct comparisons of mutations in these genes confirmed that protease production is increased in all of these mutants but also demonstrated that the impact of mutating *sarA* on protease production is greater than the impact of mutating any other regulatory locus in both LAC and UAMS-1. In fact, the only regulatory mutant in this group of mutants that exhibited protease and biofilm phenotypes even approaching those observed in *sarA* mutants was *rot*, which has also been proposed as a therapeutic target in biofilm-associated infection [159], but while this was true in LAC, it was not true in UAMS-1 [122]. Although the abundance of a captured protein and its function are not necessarily directly correlated, this is consistent with the observation that SarA was the most abundant protein captured by all four protease-associated DNA baits [122].

Conversely, mutation *mgrA* and *rot* in UAMS-1 decreased virulence in our osteomyelitis model [20]. However, this was not the case for either mutant in LAC [20], once again emphasizing the importance of considering the diversity among clinical isolates of S. aureus. To our knowledge, there are no reports describing an inhibitor of *rot*, but there is a recent report that described an inhibitor of *mgrA* [160], although these strain-dependent virulence differences argue against these regulatory loci as viable targets. Indeed, a *sarA* mutant was also the only mutant that exhibited reduced virulence in both LAC and UAMS-1 in our osteomyelitis model [20]. The protease and biofilm phenotypes of *sarA* mutants were also evident irrespective of the functional status of any of these other regulatory loci [19]. The same is true of the *msaABCR* operon [21], which is a regulatory element upstream of *sarA* [161]. Specifically, mutation of the *msa* operon (*msaABCR*) results in reduced production of SarA, increased protease production, and decreased biofilm formation [161,162], but to a limited extent by comparison to mutation of *sarA* [21]. This is consistent with the observation that mutation of *msa* limits but does not abolish SarA production. Mutation of *msa* in LAC contributes to the pathogenesis of implant-associated osteomyelitis and potentially important phenotypes including persister cell formation, cell wall formation, autolysis, and capsule production, and while there is phenotypic overlap with *sarA* mutants the extent to which this can be attributed to the impact of *msa* on *sarA* expression is unclear [163,164,165,166]. This is also another example of the need to consider diverse clonal lineages of *S. aureus* in that, unlike mutation of *sarA*, the impact of mutating *msa* on virulence in a murine bacteremia model varies in a strain-dependent manner [21].

## 17. What About *S*. *epidermidis?*

*S. aureus* is the most common cause of the most serious orthopaedic infections, but in the context of frequency, it is rivaled by *S. epidermidis*, particularly in the specific context of implant-associated infection. *S. epidermidis* encodes a SarA homolog with 84% homology to the *S. aureus* protein [167], but the impact of *sarA* on biofilm formation in *S. epidermidis* is less clear. Specifically, there is a report concluding that mutation of *sarA* limits biofilm formation owing to decreased expression of the *icaADBC* operon and reduced production of PIA [168], but there is also a report concluding that mutation of *sarA* enhances rather than limits biofilm formation owing to increased production of the extracellular matrix binding protein (Embp) and increased production of the metalloprotease SepA, with the latter being responsible for increased processing of autolysin E (AtlE) resulting in increased release of eDNA [169]. These reports utilized different strains of *S. epidermidis*, which could account for this discrepancy, but this remains to be determined.

## 18. Summary and Conclusions

While it would be preferable to identify an anti-biofilm target that is conserved in all of the approximately 44 different staphylococcal species, *S. aureus* remains the primary staphylococcal pathogen in all forms of human infection, if not in number, then certainly in severity. The collective results discussed above support the hypothesis that *sarA* is a viable therapeutic target in the important clinical context of *S. aureus* biofilm-associated infections, including osteomyelitis, based on the following observations: (1) mutation of *sarA* increases protease production to a greater degree than mutation of any other *S. aureus* regulatory locus identified to date [122]; (2) mutation of *sarA* limits biofilm formation more than mutation of any other *S. aureus* gene [19]; (3) the impact of *sarA* on both of these phenotypes is evident irrespective of the functional status of other regulatory loci including *agr* [52]; (4) the limited ability of *sarA* mutants to form a biofilm is correlated with increased antibiotic susceptibility [10,12,75,76]; (5) the impact of *sarA* on these phenotypes is evident in diverse clinical isolates including both methicillin-resistant and methicillin-susceptible strains [19,20,55,112,122], and (6) *sarA* mutants are attenuated in animal models of sepsis, septic arthritis, pneumonia, endophthalmitis, implant-associated infection, and osteomyelitis [19,20,52,55,102,115,124,170,171,172]. This all provides support for *sarA* as a preferred therapeutic target in biofilm-associated *S. aureus* if not *S. epidermidis* infections. Additionally, given the global impact of increased protease production on the *S. aureus* proteome, a *sarA* inhibitor might also prove useful in the clinical context of acute, toxin-mediated infections, and even in the context of spontaneous agr mutants that arise in vivo. The cumulative results also suggest that *sigB* and *xerC* may be viable alternatives that warrant additional consideration.

The relationship between increased protease production and decreased biofilm formation in vitro is clearly cause-and-effect, and in several animal models, it has been confirmed that increased protease production contributes significantly to the reduced virulence of *sarA* mutants. To some degree, this is counterintuitive in that these same proteases serve important functions on behalf of *S. aureus* that include tissue invasion, nutrient acquisition, and destruction of host defenses [173,174,175,176,177,178,179,180]. The demonstration that increased protease production defines most if not all clinically relevant phenotypes of *sarA* mutants does not contradict this conclusion. Rather, it demonstrates that protease production must be limited to a level that benefits *S. aureus* without compromising its own proteome. On the reverse side of this regulatory coin, eliminating protease production in LAC itself enhances virulence in murine models of both sepsis and osteomyelitis [52,147,148]. To the extent that hyper-virulence is not the goal of an opportunistic pathogen, this illustrates that *S. aureus* needs to produce these proteases, but that the ability to limit their production as a means of post-translational regulation is equally important.

Based on this, we propose that *sarA*, *sigB*, and potentially *xerC* are the most viable anti-biofilm targets identified to date. We also propose that *sarA* is the most validated of these targets for the reasons detailed above. Whether *sigB* or *xerC* have advantages over *sarA* will require in vivo experiments directly comparing the impact of these regulatory loci alone and in combination with each other using a validated animal model of biofilm-associated infection, preferably with and without concurrent antibiotic therapy. Regardless of the outcome of these studies, we suggest that the cumulative data justify targeted efforts to identify an effective inhibitor of *sarA* regulatory functions that can perhaps be exploited to prophylactic and/or therapeutic advantage. In fact, the literature would suggest that such inhibitors have already been identified. Indeed, a PubMed search using the search terms “staphylococcus aureus biofilm inhibitor” identified 624 papers, 21 of which include the search term “*sarA*”. Substituting “*sarA*” with “*xerC*” did not identify any publications, while substituting “*sarA*” with “*sigB*” identified 6. In total, 3 of these manuscripts are among the 21 “*sarA*” manuscripts because they collectively conclude that ascorbic acid (vitamin C), candesartan (Atacand), diclofenac (Flector, Cambia, Dyloject, Zipsor, Zorvolex), dexamethasone (DMSO, Maxidex, Ozurdex, DexPak), domperidone (Motilium), miconazole and sodium bicarbonate inhibit the functions of multiple regulatory loci including *sarA*, *sigB*, and *agr* [181,182,183]. The level of inhibition for all three regulatory loci was generally below 70%, but it was phenotypically evident in several respects including limiting biofilm formation [181,182,183]. To the extent that comparisons can be made between independent reports, none of these compounds inhibited biofilm formation to a degree comparable to that observed in *sarA* mutants. Most also limited rather than increased protease production, suggesting that the mechanistic basis of inhibition may involve factors other than the impact of these compounds on expression of *sarA* or *sigB*. Several were also found to inhibit expression of the *ica* operon, suggesting that their impact on biofilm formation may be due to reduced production of PIA.

When using tryptic soy broth (TSB) to assess biofilm formation, supplementation of the medium with salt and glucose enhances biofilm formation, as does coating the substrate with human plasma [67,68]. None of these factors were used in the assays used to assess biofilm formation in the presence of the drugs cited above. Using an assay that utilizes both supplementation and plasma coating has been proven to yield a *sarA* biofilm phenotype consistent with phenotypes observed in vivo [10,52,53,55,56,122,124,170], we made direct comparisons of 19 putative inhibitors of *S. aureus* biofilm formation by comparison to a *sarA* mutant. Only one of these (telithromycin) was found to inhibit biofilm formation in both LAC and UAMS-1 to a degree comparable to their isogenic *sarA* mutants [184]. Telithromycin is a bacteriostatic ketolide marketed under the trade name Ketek. It was approved for use in the United States in 2004 but based on a concerning safety profile related to liver toxicity, the only current clinical indication is community-acquired pneumonia [185]. This does not rule out its potential as a biofilm inhibitor since inhibition was observed at a concentration (0.12 and 0.49 µM in UAMS-1 and LAC, respectively) below that required to limit growth [184], thus suggesting low-dose telithromycin might be useful to limit biofilm formation and increase the efficacy of other antibiotics in biofilm-associated infections, but determining this in the context of telithromycin or any of the drugs discussed above will require in vivo validation in a relevant animal model.

In this respect, it is important to note that the *sarA* mutants employed in our comparisons are null mutants and the degree to which the abundance of SarA must be limited to see a corresponding decrease in biofilm formation, increase in antibiotic susceptibility, and decrease in virulence remains unknown. Nevertheless, it is at best uncertain whether an effective, clinically useful inhibitor of *S. aureus* biofilm formation has been identified, particularly given the mechanistic differences used to evaluate potential inhibitors in vitro. However, we propose that bringing modern technologies to bear on this increasingly important clinical problem in the specific context of *sarA* has tremendous therapeutic promise, particularly since the phenotypes of *sarA* mutants that are critical to in vivo phenotypes in biofilm-associated *S. aureus* infections are evident, even in the spontaneous *agr* mutants known to arise in vivo during human infection. We also propose that *sigB* and perhaps *xerC* warrant further exploration in this regard.

## Data Availability

No new data were created or analyzed in this study.
